# Transarterial Chemoembolization Versus Transarterial Radioembolization in Hepatocellular Carcinoma: A Systematic Review and Meta-Analysis of Real-World and Clinical Trial Evidence

**DOI:** 10.3390/cancers18121985

**Published:** 2026-06-18

**Authors:** Priyanka Gogna, Cindy Wang, Dex Underwood, Mufiza Farid-Kapadia, Manikanta Dasari, Tushar Pyne, Nilanjan Sinha, Heide A. Stirnadel-Farrant, Stephen J. Valerio

**Affiliations:** 1 AstraZeneca, Mississauga, ON L4Y 1M4, Canada; mufiza.kapadia@astrazeneca.com; 2 AstraZeneca, Gaithersburg, MD 20878, USA; cindy.wang3@astrazeneca.com (C.W.); dex.underwood@astrazeneca.com (D.U.); stephen.valerio@astrazeneca.com (S.J.V.); 3 Indence Research Private Limited, Kolkata 700156, India; mdasari@indencehealth.com (M.D.); tpyne@indencehealth.com (T.P.); nsinha@indencehealth.com (N.S.); 4 AstraZeneca, Cambridge CB2 0AA, UK; heide.stirnadel-farrant@astrazeneca.com

**Keywords:** transarterial chemoembolization, TACE, transarterial radioembolization, TARE, hepatocellular carcinoma, HCC

## Abstract

(1) Background: This systematic literature review (SLR) and meta-analysis evaluated the comparative clinical effectiveness and safety of transarterial radioembolization (TARE) versus transarterial chemoembolization (TACE) in patients with hepatocellular carcinoma (HCC). (2) Methods: Eligible studies were identified through an SLR following PRISMA guidelines using a predefined PICOS framework. PubMed and Embase were searched (2015–2025) for randomized controlled trials (RCTs) and observational studies comparing TARE and TACE. Meta-analyses were conducted for outcomes, including overall survival (OS), objective response rate (ORR), progression-free survival (PFS), and adverse events (AEs). Analyses were conducted for the overall population and predefined subgroups of interest. (3) Results: Among 1464 studies identified through database searches, 25 studies were selected for meta-analysis comprising 8146 patients with HCC. No significant difference was observed between TARE and TACE in 14 studies evaluating OS (HR: 0.99; 95% CI 0.70–1.39) or 13 studies evaluating ORR (RR: 0.94; 95% CI 0.84–1.05). Four studies reporting meta-analyzable PFS outcomes numerically favored TARE, although the difference was not statistically significant (HR: 0.54; 95% CI 0.29–1.01). Rates of any-grade and grade ≥3 AEs were comparable between the two arms. The results from subgroup analyses remained consistent with the overall findings. (4) Conclusions: TARE and TACE demonstrate comparable clinical outcomes in patients with HCC. Additional studies within specific, clinically meaningful subgroups of HCC patients will be valuable to further clarify the comparative effectiveness and safety of TARE and TACE in these populations.

## Simple Summary

Hepatocellular carcinoma (HCC) is frequently treated with locoregional therapies, yet the comparative effectiveness of transarterial chemoembolization (TACE) and transarterial radioembolization (TARE) has remained uncertain. We conducted a PRISMA-guided systematic literature review and meta-analysis of randomized and observational studies published between 2018 and 2025. Across 14 studies evaluating overall survival, the pooled hazard ratio was 0.99 (95% CI: 0.70–1.39), indicating no meaningful difference between modalities. Similarly, overall response rate (13 studies; RR: 0.94) and progression-free survival (4 studies; HR: 0.54) showed no statistically significant divergence. Safety profiles were likewise comparable, with grade ≥3 adverse events (seven studies; RR: 0.67) and any adverse events (eight studies; RR: 0.91) demonstrating no significant between-group differences. These findings suggest that TACE and TARE are broadly comparable therapeutic options in HCC management and underscore the importance of individualized treatment selection based on patient-specific factors rather than assumed superiority of either approach.

## 1. Introduction

Hepatocellular carcinoma (HCC) is the most common primary liver malignancy and remains a leading cause of cancer-related mortality worldwide [1]. HCC is commonly staged using the Barcelona Clinic Liver Cancer (BCLC) system, which categorizes the disease into early stage (single or small nodules), intermediate stage (multiple nodules), and advanced stage (vascular invasion or extrahepatic spread) [2]. Patients with early-stage disease typically undergo curative-intent treatments, such as surgical resection, local ablation, and liver transplantation, whereas those with intermediate- and advanced-stage disease are generally managed with locoregional therapies (LRTs) or systemic therapies [3,4].

Transarterial chemoembolization (TACE) and transarterial radioembolization (TARE) are widely used LRT options for the treatment of HCC [5]. TACE has long been the standard of care for unresectable intermediate-stage HCC, with conventional TACE (cTACE) and drug-eluting bead TACE (DEB-TACE) being the most used techniques [6]. With respect to chemotherapeutic agents, doxorubicin remains the most widely used drug across TACE variants, although other agents, including cisplatin, epirubicin, mitomycin C, and miriplatin, have also been employed [7]. Beyond cTACE and DEB-TACE, bland transarterial embolization (TAE) represents another locoregional approach, utilizing embolic agents without cytotoxic drugs; recent meta-analyses have reported comparable survival outcomes between TAE and chemotherapy-based TACE, raising unresolved questions about the incremental oncological contribution of the chemotherapy component in locoregional therapy [8]. TACE is typically delivered to control multifocal or more extensive intrahepatic disease in patients with HCC [9]. The therapeutic intent of TARE depends on the dose and delivery technique, with high-dose radiation segmentectomy (segmental delivery > 400 Gy) serving as a curative-intent option in patients with early-stage disease and non-segmental delivery being used as a palliative option in intermediate- and advanced-stage HCC [10,11]. In the palliative setting, TARE is often used in patients with macrovascular invasion, including portal vein tumor thrombosis (PVTT), where embolization-based approaches such as TACE may be less suitable [12]. In this context, TARE has been associated with favorable tolerability, a lower incidence of post-embolization syndrome, and encouraging outcomes in selected patients with PVTT [13,14]. In some regions, including the United States, France, and South Korea, TARE is increasingly used as a primary intra-arterial therapy rather than being reserved solely for patients unsuitable for TACE, reflecting its broader clinical adoption [5,15,16,17]. Notably, the real-world delivery of these locoregional techniques is far from uniform. Procedural parameters, including catheter selectivity (lobar, segmental, or subsegmental), volume and preparation of chemotherapeutic and embolic agent, dosimetry strategies, and institutional administration protocols, vary considerably across institutions and have evolved over time, adding further heterogeneity to study results [18,19,20].

The choice of treatment between TARE and TACE may depend on a variety of factors, such as tumor characteristics, overall liver function, and treatment objectives. However, comparative clinical outcomes of TARE versus TACE in patients with HCC remain uncertain, and there are currently no standard guidelines on patient characteristics that drive the decision between the two LRT modalities. Technical aspects of LRT delivery, including catheter positioning, embolic agent selection, and dosimetry strategy, are influenced by patient- and tumor-specific factors and should be optimized by the treating interventional radiologist. However, the multidisciplinary tumor board plays a critical role in unbiased case selection by integrating hepatology, medical oncology, surgical, radiologic, and interventional radiology perspectives to determine whether LRT is appropriate and where it best fits within the overall treatment pathway. This multidisciplinary review helps ensure that patients are selected based on disease stage, liver function, tumor burden, available alternatives, and treatment intent, rather than on procedural availability or a single-specialty perspective. Therefore, structured tumor board review remains essential to support individualized, unbiased allocation among competing locoregional and systemic treatment strategies [21,22,23].

Evidence from adequately powered and well-designed prospective head-to-head trials directly comparing these approaches is limited. Although prior systematic reviews and meta-analyses have established an evidence base, they are subject to limitations, including inconsistent outcome definitions and reporting, differences in patient selection, variations in methods of treatment delivery and differences in baseline disease severity [24,25,26,27]. The present systematic literature review (SLR) and meta-analysis aimed to evaluate and compare the clinical outcomes of TARE versus TACE in patients with HCC.

## 2. Materials and Methods

An SLR was conducted to identify comparative studies evaluating the clinical outcomes of TARE versus TACE in patients with HCC. Eligible studies were identified and subsequently assessed for feasibility of quantitative synthesis. When sufficient and methodologically comparable data were available, meta-analyses were performed to estimate pooled treatment effects. Prespecified subgroup analyses were conducted to explore heterogeneity and assess the consistency of pooled estimates across study subsets. The protocol for this review was developed prior to study initiation but was not registered or publicly available.

### 2.1. Study Selection and Eligibility Criteria

The review was conducted in accordance with the Preferred Reporting Items for Systematic Reviews and Meta-Analyses (PRISMA) guidelines [28] [Table S1]. Eligibility criteria were defined using the population, intervention, comparators, outcomes, and study design (PICOS) framework (Table 1). As TACE and TARE are established LRT options for patients with HCC across disease stages, the present study included HCC patients treated with these modalities. To account for the heterogeneity arising from the inclusion of a broad patient population, prespecified subgroup analyses were conducted to evaluate the effectiveness and safety of TARE versus TACE in relevant sub-populations or study subsets of interest. Direct comparative studies published between 2015 and 2025 evaluating TARE versus TACE in adult patients with HCC were included. Randomized controlled trials (RCTs) and non-randomized comparative studies were eligible. Single-arm studies evaluating only one LRT modality, and studies assessing sequential treatment strategies (e.g., TACE followed by TARE or where TACE/TARE was administered alongside other LRTs or systemic treatments) were excluded to minimize treatment contamination and heterogeneity. In studies with more than two treatment arms, only the TARE and TACE arms were considered in the analysis. No language restrictions were applied to maximize the identification of relevant publications.

A systematic search of PubMed and Embase was conducted according to the predefined PICOS framework to identify studies published between 1 January 2015 and 9 October 2025. The search strategy combined controlled vocabulary (MeSH and Emtree terms) and free-text keywords related to HCC and transarterial therapies, including “TACE”, “TARE”, “SIRT”, “transarterial chemoembolization”, “transarterial radioembolization”, and “selective internal radiation therapy” [Tables S2 and S3]. Reference lists of relevant reviews and included studies were also screened to ensure completeness.

Screening was performed in two sequential steps. Titles and abstracts were independently reviewed by two reviewers (NS and TP) to identify potentially eligible studies, followed by full-text assessment to determine final inclusion. Discrepancies at both stages were resolved through discussion or adjudication by a third reviewer (MD). Inter-reviewer agreement was evaluated using Cohen’s kappa statistic.

### 2.2. Quality Appraisal of Studies

Risk of bias (RoB) assessment was conducted to evaluate methodological study quality of the included studies and identify potential sources of bias related to study design, outcome measurement, selective reporting, and confounding factors. RoB was independently evaluated by two reviewers (NS and TP). RCTs were assessed using the Cochrane Risk of Bias 2.0 tool [29], and non-randomized studies were appraised using the Newcastle–Ottawa Scale [30]. Discrepancies were resolved by consensus or adjudication by a third reviewer (MD). Publication bias was assessed using funnel plots and Egger’s regression test [31] when sufficient studies were available.

### 2.3. Outcomes

The outcomes pre-specified for meta-analysis included OS, ORR, PFS, time to treatment progression (TTP), grade ≥3 adverse events (AEs), any-grade AEs, and treatment-related mortality.

### 2.4. Meta Analysis

All analyses were conducted using R software (version 4.5.1). Time-to-event outcomes were synthesized using hazard ratios (HRs), and dichotomous outcomes were pooled using risk ratios (RRs), each with 95% confidence intervals (CIs). When HRs for OS or PFS were not reported, they were estimated from reconstructed individual patient data derived from Kaplan–Meier (KM) curves using the method of Guyot et al., provided the proportional hazards assumption was satisfied [32].

Random-effects meta-analyses were conducted using the DerSimonian-Laird method to account for anticipated heterogeneity across studies; therefore, a random-effects model was preferred over a fixed-effect model [33]. Meta-analysis was performed only when at least three studies reported for a given outcome. For categorical outcomes, studies with sparse event data (fewer than two events in any arm) were excluded from pooling and were summarized narratively [34]. Statistical heterogeneity was assessed using the I^2^ statistic, with values of approximately 25%, 50%, and 75% interpreted as low, moderate, and high heterogeneity, respectively [35]. All statistical tests were two-sided, and a *p*-value < 0.05 was considered statistically significant.

Prespecified subgroup analyses were conducted to investigate potential sources of heterogeneity and evaluate the robustness of the pooled estimates. A subgroup analysis examined differences according to study design (i.e., RCTs and RWE studies). Additional subgroup analyses were performed based on the geographic location of studies, comparing Western regions (North America and Europe, e.g., USA, Germany, Belgium) with those conducted in non-Western regions (Asia–Pacific and Africa: China, Japan, South Korea, etc.) to account for regional differences in clinical practice and guideline recommendations. Studies were also stratified by publication era (before-2021 vs. 2021 and onwards), reflecting the period during which personalized dosimetry for TARE began gaining wider adoption in the US [20]. Treatment-naïve status was analyzed to compare outcomes in patients receiving TACE or TARE as first-line locoregional therapy versus those previously treated.

A subgroup analysis was further performed restricting the analysis to studies demonstrating adequate baseline comparability between the treatment arms. These included studies employing appropriate adjustment for key tumor- and disease-related variables (e.g., tumor size and exclusion of PVTT) using statistical techniques such as multivariable regression, inverse probability of treatment weighting (IPTW), or propensity score matching (PSM), and that excluded crossover between TACE and TARE during follow-up.

Differences between subgroups were assessed using the Cochran Q test for subgroup heterogeneity. Additional subgroup analyses based on Child-Pugh (CP) classification, ALBI grade, vascular invasion (Vp) classification, and tumor characteristics, were planned; however, meta-analysis was not feasible due to the limited number of studies reporting these outcomes.

## 3. Results

### 3.1. Selection of Studies

**Figure 1:**
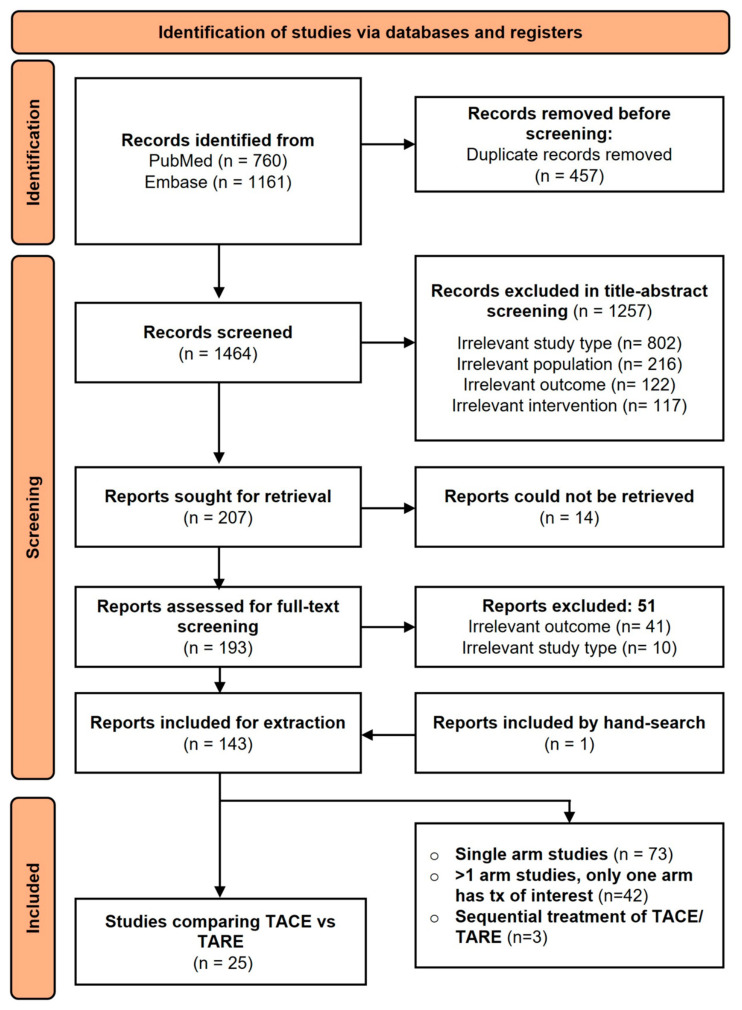
PRISMA flow diagram. Abbreviations: Embase: Excerpta Medica Database, TACE: Transarterial chemoembolization, TARE: Transarterial radioembolization, tx: Treatment.

Database searches of PubMed and Embase identified 1464 publications. Based on the pre-defined PICOS criteria, 143 studies were found to be relevant. Inter-reviewer agreement (κ) was 0.82 for title-abstract screening and 0.83 for full-text screening. Twenty-five out of the 143 selected studies reported a direct comparison of TACE and TARE with relevant outcomes and were included in the final evidence synthesis [36,37,38,39,40,41,42,43,44,45,46,47,48,49,50,51,52,53,54,55,56,57,58,59,60]. The remaining studies were excluded because they were either single-arm studies (*n* = 73), multi-arm studies reporting only one relevant TACE or TARE arm (*n* = 42), or studies evaluating sequential TACE or TARE treatment strategies (*n* = 3). The flow of studies through the different phases of the SLR is summarized in the PRISMA flow diagram (Figure 1).

### 3.2. Overview of Included Evidence

Among the 25 studies included in the final evidence base, 17 were retrospective observational studies [36,37,38,39,40,42,47,49,50,51,52,53,56,57,58,59,60], 4 were prospective observational studies [41,44,45,46], and 4 were RCTs [43,48,54,55]. The included studies were conducted across multiple geographic regions, including the USA [36,37,39,41,46,51,52,55,58], Asia–Pacific (APAC) [47,49,53,55,56,59,60], Europe [38,40,42,43,45,48,50,54,57], and one study from Africa [44].

A total of 8146 patients with HCC were included across all studies. The mean age ranged from 58 to 70 years. Patient distribution was reflective of the heterogeneity of the embolization-eligible HCC population. Across studies, the proportion of females ranged from 5.6% to 32.1%. Nine studies reported Eastern Cooperative Oncology Group Performance Status (ECOG PS), with 80% to 90% of patients classified as ECOG PS 0-1 [39,43,46,47,48,51,52,56,60]. Liver disease characteristics were reported inconsistently across studies. Cirrhosis prevalence ranged from 31.5% to 100%, and PVTT ranged from 1% to 100% where reported. ALBI grades were predominantly grade 1–2 (84–100%). Subsequent liver transplantation ranged from 0% to 80.9% across treatment arms.

Treatment delivery approaches varied considerably across studies, as summarized in Table 2. TACE was most commonly administered using doxorubicin (50–150 mg per session), delivered via lipiodol emulsion or drug-eluting beads. Other reported differences included the embolic platform, number of treatment sessions, and TACE subtype, including conventional TACE (*N =* 19 studies), DEB-TACE (*N =* 5 studies), DSM-TACE (*N =* 1 study), and DEE-TACE (*N =* 1 study). Similarly, TARE approaches varied across studies, with differences in Y90 TARE (N = 19 studies)/SIRT platforms (*N* = 6 studies), treatment extent, and dosimetry method. TARE dosing was guided by dosimetry, with reported absorbed doses ranging from 80 to 360 Gy [39,47,51,52,55]. No study reported TARE administered at doses exceeding 400 Gy in the context of radiation segmentectomy (expected in the curative intent setting), indicating capture of patients treated with TARE in a palliative intent setting across studies evaluated.

Regarding outcomes assessed, OS was reported in 23 studies [36,37,38,39,40,41,42,43,44,45,46,47,49,50,51,52,53,54,55,56,57,58,59], ORR in 14 studies [36,37,39,43,44,47,48,49,52,53,55,56,58,59], PFS in 9 studies [37,38,43,47,48,49,52,53,54], grade ≥3 AEs in 11 studies [36,37,39,43,47,48,51,52,53,55,56], any-grade AEs in 17 studies [36,37,38,39,43,44,45,47,48,49,51,52,53,55,56,58,59], TTP in 5 studies [43,44,54,55,60], and treatment-related mortality in 5 studies [42,43,45,50,58]. An overview of study characteristics and reported outcomes is provided in Table 2. RoB assessment was conducted for RWEs and RCTs separately, and the results are presented separately in Tables S4 and S5.

### 3.3. Meta-Analysis

#### 3.3.1. Overall Survival

**Figure 2:**
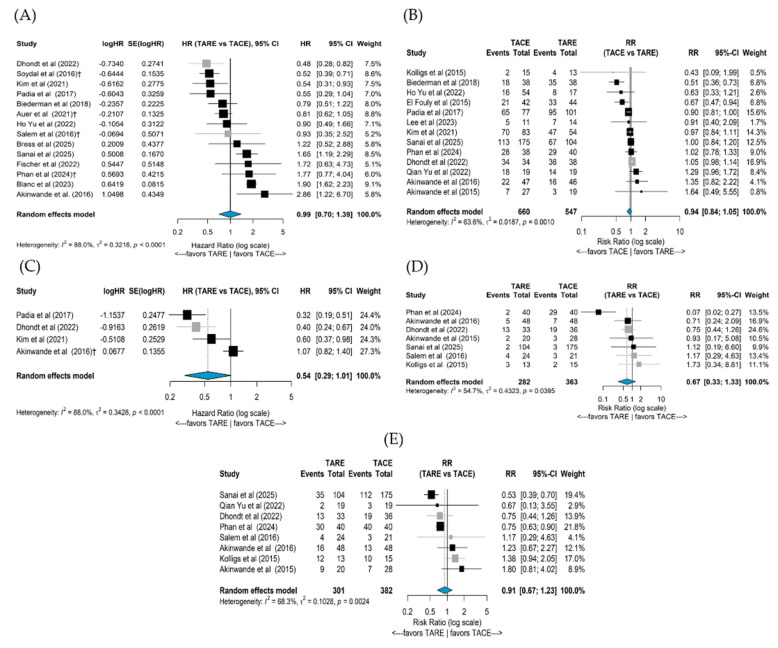
Results of overall meta-analysis for (**A**) overall survival [37,38,39,40,41,43,45,47,52,53,55,56,57,59], (**B**) objective response rate [36,37,39,43,44,47,48,49,52,53,56,58,59], (**C**) progression-free survival [37,43,47,52], (**D**) grade 3+ AEs [36,37,43,48,53,55,56], and (**E**) any AEs [36,37,43,48,53,55,56,58]. Studies marked with ‘†’ indicate that the HR was derived from reconstructed IPD after confirming that the PH assumption was not violated; Effect sizes shown in grey represent RCTs; the remaining studies are observational. Pooled effect size <1 implies TARE is favored and >1 implies TACE is favored. Abbreviations: AE: adverse event; CI: confidence interval; IPD: individual patient data; HR: hazard ratio; PH: proportional hazard; RR: risk ratio; SE: standard error; TACE: transarterial chemoembolization; TARE: transarterial radioembolization.

Fourteen studies out of twenty-five included studies were eligible for meta-analysis of OS [37,38,39,40,41,43,45,47,52,53,55,56,57,59]. Eleven studies were excluded due to the absence of any OS outcomes, lack of non-reported HRs, absence of KM curves, or violation of the PH assumption following KM reconstruction. Among the 14 eligible studies, 10 reported HRs directly, while HRs were reconstructed using individual patient data in 4 studies. The pooled analysis demonstrated no significant difference in OS between TARE and TACE (pooled HR: 0.99; 95% CI: 0.70–1.39; Figure 2A). Ten out of fourteen studies included for meta-analysis had low risk of bias (Table S4). No publication bias was detected (*p* = 0.53; Figure S6). Substantial heterogeneity was observed (I^2^ = 88%), indicating considerable variability in effect estimates across studies. Prespecified subgroup analyses aimed at understanding sources of potential heterogeneity showed consistent results across study subsets, with no significant differences between TARE and TACE (Table 3; Figure S1). In some subgroup analyses, heterogeneity decreased, including US-based studies (I^2^ = 60.9%), studies with controlled baseline characteristics (I^2^ = 58.2%), and treatment-naïve populations (I^2^ = 47.2%).

Meta-analyses by CP class, ALBI grade, Vp status, and tumor characteristics were not feasible due to limited and inconsistent reporting. Among patients with CP-A liver function, several studies reported longer OS with TACE compared to TARE, including Sanai et al. (HR: 1.82, 95% CI: 1.12–2.42, TACE vs. TARE) [56], Hickey et al. (median OS: 21 months with TACE vs. 15.7 months with TARE) [46], and McDevitt et al. (median OS: 10 months with DEB-TACE vs. 8.2 months with Y-90 TARE) [51]. Ho Yu et al. observed no significant difference between TARE and TACE (HR: 0.90; 95% CI: 0.50–1.70) [59]. Findings in CP-B patients were inconsistent, with one study favoring TACE [46], and another favoring TARE [51]. Sanai et al. reported comparable OS between TARE and TACE (HR: 1.82; 95% CI: 0.95–3.45) in CP-B patients [56].

For ALBI grade, Hickey et al. reported longer median OS with cTACE across ALBI grades 1–3 [46]. In patients with macrovascular invasion (Vp3/Vp4 PVTT), Kim et al. reported no significant difference in OS between TARE and TACE (HR: 0.44; 95% CI: 0.18–1.10) [47]. Overall, findings across these subgroups were heterogeneous and limited by the small number of studies.

#### 3.3.2. Objective Response Rate

Thirteen studies reported ORR [36,37,39,43,44,47,48,49,52,53,56,58,59]. The pooled analysis showed no statistically significant difference between TARE and TACE (RR: 0.94; 95% CI: 0.84–1.05; Figure 2B). Eight out of thirteen studies included for meta-analysis had low RoB (Table S4). No publication bias was detected (*p* = 0.41; Figure S6). Moderate between-study heterogeneity was observed (I^2^ = 63.6%). Subgroup analyses of ORR (Figure S2) showed reduced heterogeneity when studies were stratified by publication year (I^2^ = 0% for studies published 2021 and onwards) and geographic region (I^2^ = 19.3% in non-Western settings). However, pooled effect estimates remained consistent across analyses, indicating no clinically meaningful difference between treatment modalities.

#### 3.3.3. Progression-Free Survival

Four studies were eligible for meta-analysis of PFS [37,43,47,52]. The pooled analysis numerically favored TARE over TACE (HR: 0.54; 95% CI: 0.29–1.01; Figure 2C), although the difference did not reach statistical significance. Three of the four studies included in the meta-analysis had low RoB (Table S4). Publication bias was detected (*p* = 0.006; Figure S2); however, tests for publication bias may be unreliable when fewer than ten studies are included. Substantial heterogeneity was observed across studies (I^2^ = 88%). Only three predefined subgroup analyses were feasible owing to the limited number of studies reporting PFS (Figure S3). No significant differences between TARE and TACE were observed across these analyses, and heterogeneity remained high.

#### 3.3.4. Time-To Progression

TTP was reported in five studies [43,44,54,55,60], but insufficient reporting of HRs limited quantitative synthesis to two studies, rendering meta-analysis infeasible. Two RCTs demonstrated significantly longer TTP with TARE compared with DEB-TACE or cTACE (HR: 0.36 in Dhondt et al.; HR: 0.12 in Salem et al.) [43,55]. Pitton et al. reported longer median TTP with SIRT compared with DEB-TACE (12.4 vs. 11.2 months) [54]. In contrast, Hirsch et al. and El Fouly et al. reported longer median TTP with DEB-TACE or conventional TACE compared with TARE [44,60].

#### 3.3.5. Grade ≥3 Treatment-Related Adverse Events

Seven studies reported grade ≥ 3 treatment-related AEs [36,37,43,48,53,55,56]. The pooled RR suggested a non-significant trend toward fewer severe AEs with TARE compared with TACE (RR: 0.67; 95% CI: 0.33–1.33; Figure 2D). Three of the seven studies included in the meta-analysis had a low risk of bias (Table S4). No publication bias was detected (*p* = 0.8; Figure S6). Between-study heterogeneity was moderate (I^2^ = 54.7%). Across predefined subgroup analyses, no significant differences were observed between TACE and TARE (Table 3; Figure S4). However, statistical heterogeneity decreased when analyses were stratified by geographic region (I^2^ = 0% in western studies; I^2^ = 0% in US-based studies), publication era (I^2^ = 0% in studies published before 2021) and study design (I^2^ = 0% in RCT). A notable reduction in heterogeneity, from 52% to 5%, was observed when the study by Phan et al. was excluded [53]. This study included patients with large unresectable tumors (>8 cm) and applied a personalized dosimetry approach. In addition, several laboratory-based AEs were reported more frequently in the TACE group than in the TARE group, which may have contributed to the observed heterogeneity. The thresholds used to define these laboratory-based AEs were also not clearly described, further limiting the comparability with other studies.

#### 3.3.6. Adverse Events of Any Grade

Eight studies contributed data on any-grade AEs [36,37,43,48,53,55,56,58]. Meta-analysis demonstrated no significant difference between TARE and TACE (RR: 0.91; 95% CI: 0.67–1.23; Figure 2E). Four of eight studies included in the meta-analysis had low RoB (Table S4). No publication bias was detected (*p* = 0.36; Figure S6). Substantial heterogeneity was observed (I^2^ = 68.3%), reflecting differences in study populations and reporting practices. Stratified analyses led to reduced heterogeneity when studies were grouped by geographic region (I^2^ = 2% in Western studies and I^2^ = 0% in US-based studies) and publication era (I^2^ = 0% in before-2021 studies and I^2^ = 31.2% in 2021 and onwards studies) (Figure S5). Heterogeneity appeared to be largely influenced by two studies [53,56]. In Sanai et al., patients in the TARE group had larger tumors and treatment allocation was guided by treatment phenotype, resulting in baseline differences among treatment arms that were not fully adjusted for [56].

#### 3.3.7. Treatment-Related Mortality

Five studies reported treatment-related mortality [42,43,45,50,58]. However, only two met the predefined criterion of at least two events per treatment arm, precluding quantitative pooling. Findings were therefore summarized descriptively. Across individual studies, treatment-related mortality was generally lower among patients treated with TARE. Fischer et al. and Massani et al. each reported two procedure-related deaths in TACE-treated patients and none in TARE-treated patients [45,50]. Similar patterns were observed in Qian Yu et al. (four deaths in the TACE + ablation vs. three in the TARE) [58], Dhondt et al. (three deaths with DEB-TACE vs. one with TARE) [43], and Craciun et al. (one postoperative death following TACE vs. none with SIRT) [42].

## 4. Discussion

To the best of our knowledge, this SLR and meta-analysis represents the first comprehensive contemporary synthesis directly comparing TACE and TARE across HCC populations, integrating evidence from both RCTs and real-world observational studies. Rather than imposing stage-based restrictions, this analysis incorporated studies reflecting the full spectrum of clinical severity captured in routine practice. No statistically significant differences between TACE and TARE were observed in the efficacy and safety parameters assessed in this study.

TACE and TARE are established LRT modalities for unresectable HCC [6]. TACE causes hypoxia leading to tumor cell death and induces tumor necrosis through a combination of chemotherapy and embolization, whereas TARE delivers yttrium-90-loaded microspheres that provide localized radiation with minimal embolic effect. Although high-dose segmental TARE has been used as an alternative curative-intent strategy in early-stage HCC patients, it is routinely administered in a non-segmental manner for palliative disease control. This distinction has important implications for interpreting the present findings as the included studies could have differed not only in patient population and disease stage, but also in the intended therapeutic role of Y90. Although contemporary radiation segmentectomy should involve tumor-absorbed doses exceeding 400 Gy in selected curative-intent settings, none of the included studies reported Y90 TARE at this dose threshold in the context of radiation segmentectomy. Thus, the Y90 cohorts in this analysis appear to reflect broader clinical applications, including palliative disease control, downstaging, and bridging, rather than modern ablative-dose-radiation segmentectomy alone. Consequently, the present analysis primarily reflects the conventional use of these treatments, enabling a balanced comparison with standard TACE.

In the present meta-analysis, pooled analysis of 14 studies showed no significant difference in OS between TARE and TACE. To assess the influence of the markedly imbalanced cohorts (4808 TACE vs. 306 TARE patients) reported by Blanc et al., which included patients receiving concurrent systemic therapy, we performed a sensitivity analysis excluding this study from the OS meta-analysis (not reported). The pooled OS estimate remained comparable and non-significant after exclusion, suggesting that the overall finding was not driven solely by this cohort, although substantial heterogeneity persisted across studies. These findings are broadly consistent with previous meta-analyses comparing survival outcomes between the two treatments. For example, Lu et al. reported comparable OS at 3 years (*p* = 0.73) and 5 years (*p* = 0.38), although they observed higher 1-year OS with TARE (*p* = 0.02) [27]. A separate meta-analysis restricted to RCTs found no difference in 1-year OS between the two treatments (pooled OR: 1.31; 95% CI: 0.56–3.04) [61]. Similarly, other meta-analyses have also reported no significant survival advantage for either intervention. Brown et al. reported a pooled mean difference in OS of –0.55 months (95% CI: –1.95 to 3.05), while Facciorusso et al. reported a pooled HR of 0.91 (95% CI: 0.80–1.04), both indicating comparable survival between TARE and TACE [5]. In contrast, Zhang et al. reported improved survival with TARE (pooled HR: 0.74; 95% CI: 0.61–0.90) [62]. However, this analysis by Zhang et al. included only three studies limited to patients with unresectable HCC, which differs from the broader population evaluated in the present study. Overall, substantial heterogeneity was observed for OS in the present study, likely reflecting differences in patient selection, tumor burden, liver function, treatment techniques (TACE type, TARE platforms), and reporting methods. However, the findings from the meta-analysis of OS remained broadly consistent across the subgroup analyses.

Similarly, pooled analysis of ORR across 13 studies demonstrated no significant difference between treatment modalities. Moderate heterogeneity was observed, likely reflecting variation in imaging techniques, response assessment criteria, timing of evaluation, and institutional expertise. These findings are consistent with previous systematic reviews reporting broadly comparable radiologic response rates between TACE and TARE. For example, Alcantara et al. reported a pooled OR of 0.87 (95% CI: 0.41–1.82), while Facciorusso et al. reported a pooled OR of 1.22 (95% CI: 0.69–2.16) [24,25]. Two other analyses evaluating CR and PR rates also reported no significant difference between two modalities [27,62].

PFS numerically favored TARE, although the pooled estimate did not reach statistical significance and was accompanied by high heterogeneity. Notably, most individual studies (three out of four studies) included in the PFS analysis demonstrated trends favoring TARE, suggesting a possible advantage in delaying tumor progression compared with TACE. Inconsistencies between the present findings and prior meta-analyses may stem from methodological differences, including the pooling of TTP and PFS outcomes in earlier studies [25]. For instance, Facciorusso et al. reported improved 1-year PFS rate for TARE (pooled OR: 1.67; 95% CI: 1.10–2.55) [24], whereas a meta-analysis of RCTs showed no difference in 1-year PFS rates (pooled OR: 0.23; 95% CI: 0.02–2.45) [61]. Nevertheless, the limited number of eligible studies and substantial heterogeneity restricted definitive conclusions regarding progression endpoints. Interpretation of the pooled PFS estimate in the current study is limited by variations in the number of treatment cycles administered and the use of subsequent curative therapies across the included studies. For example, Padia et al. reported an average of 1.34 TACE procedures per treated tumor [52], whereas Dhondt et al. and Akinwande et al. (2016) reported approximately three to four TACE procedures per patient [37,43]. Importantly, the numerical PFS advantage observed for TARE did not translate into a corresponding difference in OS. In addition, publication bias was detected for the PFS analysis, and only a limited number of studies contributed to this outcome. Therefore, the magnitude of the observed PFS benefit with TARE may be overestimated and should be interpreted cautiously. Although publication bias tests are limited in reliability when based on a small number of studies, this finding further underscores the uncertainty surrounding the pooled PFS estimate.

Among the five studies reporting on TTP [43,44,54,55,60], insufficient reporting of HRs limited quantitative synthesis, rendering formal meta-analysis infeasible. Two single-center phase II, randomized studies demonstrated significantly longer TTP with TARE compared with DEB-TACE or cTACE, with reported HRs of 0.36 in Dhondt et al. and 0.12 in Salem et al. [43,55]. Pitton et al. also reported longer median TTP with SIRT compared with DEB-TACE [54]. Conversely, two observational studies by Hirsch et al. and El Fouly et al. reported longer median TTP with DEB-TACE or conventional TACE compared with TARE [44,60]. This directional variability is clinically relevant because TTP may be a more treatment-sensitive endpoint than OS in the setting of locoregional therapy. However, given the limited availability of HRs and heterogeneity across study designs, small patient numbers, patient selection, baseline tumor burden, and treatment delivery, TTP could not be quantitatively incorporated into the comparative efficacy conclusions. Future prospective comparative trials should further evaluate TTP as a clinically meaningful endpoint in this setting.

With respect to safety, no significant differences were observed in the incidence of grade ≥3 AEs (across seven studies) or AEs of any grade (across eight studies) between TACE and TARE. Although some individual studies reported differences in specific events, overall safety profiles were broadly comparable. Heterogeneity in safety outcomes likely reflects substantial variability in AE reporting across studies, including differences in assessment periods, follow-up duration, grading systems (e.g., varying CTCAE versions), and definitions of treatment-related versus all-cause AEs. The timing of AE evaluation ranged from 1-week post-treatment (Sanai et al.) [56] to 6 months (Dhondt et al.) [43]. For example, in the study by Sanai et al., AEs of any grade were reported in 64% of patients in the TACE arm and 33.7% in the TARE arm, despite the relatively short AE evaluation period of one week after treatment [56]. The high event rate observed in this study, particularly in the TACE arm, may partly reflect prior LRT exposure among the enrolled patients. Additional inconsistencies in reporting denominators (per patient vs. per procedure), selective reporting of severe events only, incomplete event counts, and unclear attribution further limited direct comparability and complicated quantitative pooling.

Recent advances in systemic therapy have significantly reshaped the treatment approach to unresectable HCC, with many patients now receiving systemic agents as initial therapy [63,64,65,66,67]. Despite this evolution, LRT treatments such as TACE and TARE continue to play an important role, either as stand-alone options in embolization-eligible patients or in combination with systemic therapies [68,69,70]. As both TACE and TARE remain widely used in routine practice, clarifying their relative outcomes remains important for informed clinical decision making. In this context, the present SLR and meta-analysis provides an updated assessment of the comparative clinical outcomes of TACE and TARE to inform treatment selection in contemporary practice.

This study has several strengths compared with previous systematic reviews. First, it incorporates both RCTs and real-world evidence and includes the most recent comparative literature, providing an updated synthesis of clinically relevant endpoints than earlier meta-analyses that relied on smaller or older datasets [24,62]. Importantly, the analysis reflects the broad spectrum of HCC populations represented in routine clinical practice rather than restricting inclusion to narrowly defined stage-specific cohorts, which has been a limitation of several prior reviews [5,61]. In addition, HRs reconstructed from KM curves were excluded when proportional hazards assumptions were violated, reducing potential bias. Finally, baseline comparability between treatment arms was evaluated in a dedicated subgroup analysis restricted to studies meeting predefined methodological criteria, strengthening the internal validity of the findings and addressing concerns regarding imbalance in patient characteristics reported in earlier syntheses [27].

At the same time, interpretation of these findings requires caution. Reporting in primary studies was sometimes incomplete, particularly with respect to patient-level denominators for AEs and detailed response outcomes, which limited quantitative synthesis for certain endpoints such as TTP and treatment-related mortality [42,43,44,45,50,54,55,58,60]. In addition, this analysis was restricted to direct comparative studies of TACE and TARE. Although incorporating indirect evidence through network-based approaches may provide complementary insights, such methods would introduce additional assumptions and potential heterogeneity. The predominance of retrospective observational studies introduces potential residual confounding despite statistical adjustment. Differences in patient selection, tumor burden, PVTT status, baseline liver function, study design, outcome definitions, and follow-up duration may have contributed to variability across studies. Furthermore, clinical and methodological heterogeneity, including variation in TACE techniques and number of administrations, TARE platforms, outcome definitions, and follow-up duration limits cross-study comparability. Although subgroup analyses reduced heterogeneity in selected instances, some variability remained for certain outcomes, likely reflecting differences in study design and patient characteristics across the included literature. Nevertheless, while the direction of effect estimates was generally consistent across analyses, the persistent heterogeneity between studies limits the reliability of the pooled estimates, and findings should be interpreted with appropriate caution.

## 5. Conclusions

This rigorous SLR and meta-analysis did not identify any clear clinical efficacy or safety differences in the use of TACE or TARE in patients with HCC. Although effect estimates were generally consistent across subgroup and sensitivity analyses, these findings should be interpreted with caution given the predominance of observational data, heterogeneity across included studies, and potential for residual confounding by indication. Further, well-designed comparative studies are warranted to better delineate optimal patient selection and clinical positioning of TACE and TARE. Greater standardization in the technical delivery of these procedures may help reduce heterogeneity across studies and improve the interpretability of comparative outcomes. As the therapeutic landscape of HCC continues to evolve with the earlier introduction of systemic therapies and increasing adoption of multimodal treatment strategies, these results provide important context for the role of intra-arterial locoregional therapies in contemporary practice.

## Figures and Tables

**Table 1:** PICOS criteria for inclusion of studies.

Component	Inclusion Criteria
Population	Adult patients with HCC
Intervention & comparators	Transarterial chemoembolization (TACE; conventional or drug-eluting bead); Transarterial radioembolization (TARE; selective internal radiation therapy with Y-90);
Outcomes	Efficacy or effectiveness: overall survival (OS), objective response rate (ORR), progression-free survival (PFS), time-to-progression (TTP), post-progression survival, duration of response, downstaging to curative therapy, disease control, disease specific survival. Safety outcomes: grade 3+ (TRAEs), any grade AEs, treatment-related mortality, liver-related AEs (hyperbilirubinemia, liver failure, ascites, post-embolization syndrome, cholangitis, abscess, biliary track injury, PVT, jaundice), treatment discontinuation, post-embolization syndrome, pyrexia, radiation-related toxicity [TARE only], fatigue, nausea/vomiting, fever, abdominal pain.
Study design	RCTs and observational studies (prospective or retrospective)
Time period	1 January 2015 to 9 October 2025
Country	Global
Language	Any language

Abbreviations: AEs: adverse events, HCC: hepatocellular carcinoma, PVT: portal vein thrombosis, RCT: randomized controlled trial, TRAE: treatment-related adverse events, Y-90: Yttrium 90.

**Table 2:** Summary of included evidence.

Author Year	Study Type	Country	Population	Stage	Intervention	N	Age (Mean), Years	Female (%)	Cirrhosis n (%)	PVTT n (%)	Transplant Cases [n (%)]	ECOG PS Status (%)	Embolization Agents	Outcomes Assessed
Sanai (2025) [56]	Retrospective	Saudi Arabia	Early- and intermediate-stage HCC	BCLC 0-B	TACE	175	67.3	26.86	171 (97.7%)	-	-	0–1: 85.1% 2: 14.9%	Doxorubicin	CR, PR, OS, any AEs, grade 3 or higher TRAEs, TRAEs
					TARE	104	67.9	24.04	100 (96.2%)	-	-	0–1: 91.3% 2: 8.7%	Yttrium 90 microsphere	
Bress (2025) [41]	Prospective	USA	HCC	-	TACE	144	63.7	17	98 (68.1%)	58 (55%)	-	-	Cisplatin	OS
					Y-90 TARE	90	69.7	39	61 (67.8%)	26 (25%)	-	-	Yttrium 90 microsphere	
Phan (2024) [53]	Retrospective	South Korea and Vietnam	Unresectable HCC	BCLC A-C	cTACE or DEB-TACE	89	-	21.3	-	23 (25.8%)	6 (15%)	-	Doxorubicin	CR, PR, ORR, DCR, OS, PFS, any AEs, grade 3 or higher TRAEs
					Y-90 TARE	40	-	20	-	16 (40%)	4 (10%)	-	Yttrium 90 microsphere	
Blanc (2023) [40]	Retrospective	France	Intermediate-, advanced- or terminal-stage HCC	BCLC A-C	TACE + systemic therapies	4808	70.5	-	-	-	-	-	-	OS
					TARE + systemic therapies	306	70.5	-	-	-	-	-	-	
Lee (2023) [49]	Retrospective	South Korea	Single large HCC	-	TACE	14	61.6	-	-	1 (7.1%)	1 (7.1%)	-	-	CR, PR, OS, PFS. any AEs
					TARE	13	62.8	-	-	1 (7.7%)	1 (7.7%)	-	-	
Qian Yu (2022) [58]	Retrospective	USA	Unresectable HCC	CP A-C	TACE + Ablation	29	61.5	17.2	29 (100%)	-	6 (20.7%)	-	Doxorubicin	CR, PR, ORR, OS, any AEs, treatment-related mortality
					TARE	40	69.3	30	40 (100%)	-	8 (20%)	-	Yttrium 90 microsphere	
Fischer (2022) [45]	Prospective	Germany	Early-stage HCC	BCLC A-C	TACE	57	*65*	-	53 (93%)	-	-	-	Doxorubicin, mitomycin C, and lipiodol	OS, any AEs, TRAEs, treatment-related mortality
					TARE	12	*67*	-	10 (83.3%)	-	-	-	Yttrium 90 microsphere	
Dhondt (2022) [43]	RCT	Belgium and Italy	Unresectable HCC	BCLC A-B	DEB-TACE	34	*68*	12	-	-	4 (12%)	0: 85% 1: 15%	Doxorubicin	ORR, OS, PFS, TTP, any AEs, grade 3 or higher TRAEs, treatment-related mortality
					TARE	38	*67*	13	-	-	10 (26%)	0: 90% 1: 11%	Yttrium 90 microsphere	
Ho Yu (2022) [59]	Retrospective	China	HCC	CP-A	TACE	54	*59.5*	5.6	17 (31.5%)	-	-	-	Cisplatin	CR, PR, OR, ORR, OS, any AEs
					TARE	17	*57*	23.5	8 (47.1%)	-	-	-	Yttrium 90 microsphere	
Kim (2021) [47]	Retrospective	South Korea	HCC	BCLC A-C	cTACE	84	*60*	17	84 (100%)	-	-	0: 90.5% 1: 9.9%	Doxorubicin	CR, PR, ORR, DCR, OS, PFS, any AEs, grade 3 or higher TRAEs
					Y-90 TARE	54	*58*	17	54 (100%)	-	-	0: 87% 1: 13%	Yttrium 90 microsphere	
Hirsch (2021) [60]	Retrospective	Australia	HCC	BCLC 0-D	DEB-TACE	90	67	16	83 (92.2%)	15 (16.7%)	4 (4.4%)	0: 54.4% 1: 25.6% 2: 5.6%	Doxorubicin	TTP
					SIRT (TARE)	80	62.1	11	62 (77.5%)	-	3 (3.75%)	0: 58.8% 1: 8.8% 2: 3.8%	SIRTEX© spheres	
Auer (2021) [38]	Retrospective	Germany	Multifocal HCC	BCLC B-C	DSM-TACE	18	*68.5*	22.5	18 (100%)	-	-	-	Doxorubicin	OS, PFS, any AEs, grade 3 or higher TRAEs
					SIRT (TARE)	18	*71*	16.5	18 (100%)	-	-	-	Yttrium 90 microsphere	
Craciun (2020) [42]	Retrospective	Belgium	HCC	CP-A	TACE	16	*65.1*	13	16 (100%)	-	-	-	Doxorubicin	OS, Treatment related mortality
					SIRT (TARE)	12	*62.6*	8	12 (100%)	-	-	-	Yttrium 90 microsphere	
Biederman (2018) [39]	Retrospective	USA	Early HCC	-	Segmental TACE	57	-	28.1	54 (94.7%)	-	18 (31.5%)	0: 47.4% ≥ 1: 52.6%	Doxorubicin	CR, OS, any AEs grade 3 or higher TRAEs
					SIRT	55	-	32.7	51 (92.7%)	-	8 (14.5%)	0: 69.1% ≥ 1: 30.9%	Glass microspheres	
Padia (2017) [52]	Retrospective	USA	Localized, unresectable HCC	BCLC A-D	TACE	77	*60*	-	75 (97.4%)	1 (1%)	-	0: 55.8% 1: 31.2% 2: 11.7%	Doxorubicin	CR, PR, OS, PFS, any AEs, grade 3 or higher TRAEs
					Y-90 TARE	101	*62*	-	99 (98%)	24 (18.2%)	-	0: 76.2% 1: 18.8% 2: 5%	Yttrium 90 microsphere	
Kolligs (2015) [48]	RCT	Germany	Intermediate-stage unresectable HCC	BCLC A-C	TACE	15	66.7	13.3	-	-	3 (20%)	0: 80% 1: 20%	Epirubicin	PR, CR, ORR, PFS, any AEs, grade 3 or higher TRAEs, TRAEs
					Y-90 SIRT	13	65.8	15.4	-	-	3 (23%)	0: 76.9% 1: 23.1%	Yttrium 90 microsphere	
McDevitt (2017) [51]	Retrospective	USA	Infiltrative HCC	BCLC B-C	DEE-TACE	26	*64*	15	26 (100%)	-	1 (4%)	0: 31% 1: 38% 2: 26%	Doxorubicin	OS, any AEs, grade 3 or higher TRAEs
					TARE	24	*61*	13	24 (100%)	-	-	0: 29% 1: 42% 2: 25%	Yttrium 90 microsphere	
Massani (2017) [50]	Retrospective	Italy	Unresectable HCC	BCLC A-C	TACE	82	70.3	18	82 (100%)	16 (19.5%)	-	-	Doxorubicin	OS, Treatment related mortality
					TARE	39	70.77	18	39 (100%)	10 (26%)	-	-	Yttrium 90 microsphere	
Soydal (2016) [57]	Retrospective	Turkey	HCC	BCLC B-C	TACE	40	66.15	15	-	-	-	-	Mitomycin	OS
					Y-90 TARE	40	62.28	18	-	-	-	-	Yttrium 90 microsphere	
Akinwande (2016) [37]	Retrospective	USA	Unresectable HCC	CP A-C	DEBDOX-TACE	291	*67*	26	-	30 (10%)	4 (1.3%)	-	Doxorubicin	CR, PR, DCR, OS, PFS, any AEs, grade 3 or higher TRAEs
					Y-90 TARE	67	*65*	27	-	23 (34%)	-	-	Yttrium 90 microsphere	
Salem (2016) [55]	RCT	USA	HCC	BCLC A-B	cTACE	21	*64*	24	20 (95.2%)	-	17 (80.9%)	-	Drug/lipiodol combination	CR, PR, OS, TTP, any AEs, grade 3 or higher TRAEs
					Y-90 TARE	24	*62*	29	24 (100%)	-	18 (75%)	-	Yttrium 90 microsphere	
Hickey (2016) [46]	Prospective	USA	Unresectable HCC	BCLC A-C	TACE	337	-	-	-	16 (4.7%)	-	0: 47.2% 1: 46.6% 2: 6.2%	Doxorubicin	OS
					TARE	428	-	-	-	156 (36.4%)	-	0: 54.9% 1: 38.1% 2: 7%	Yttrium 90 microsphere	
El Fouly (2015) [44]	Prospective	Egypt	Intermediate-stage HCC	BCLC B	cTACE	42	58.3	9.52	-	-	-	-	Doxorubicin	CR, PR, OS, TTP, any AEs
					Y-90 TARE	44	66.1	18.18	-	-	-	-	Yttrium 90 microsphere	
Pitton (2015) [54]	RCT	Germany	Intermediate-stage HCC	BCLC A-B	TACE	12	70.5	16.67	12 (100%)	-	1 (8.3%)	-	Doxorubicin	OS, PFS, TTP
					SIRT	12	71.8	13.33	12 (100%)	-	-	-	Yttrium 90 microsphere	
Akinwande (2015) [36]	Retrospective	USA	Unresectable HCC	CP A-C	DEBDOX-TACE	28	*66.5*	-	-	28 (100%)	-	-	Doxorubicin	CR, PR, ORR, OS, any AEs, grade 3 or higher TRAEs
					Y-90 TARE	20	*66.5*	-	-	20 (100%)	-	-	Yttrium 90 microsphere	

Abbreviations: HCC: hepatocellular carcinoma, BCLC: Barcelona Clinic Liver Cancer, TACE: transarterial chemoembolization, cTACE: conventional transarterial chemoembolization, DEB-TACE: drug-eluting bead transarterial chemoembolization, DEBDOX-TACE: drug-eluting bead doxorubicin transarterial chemoembolization, DEE-TACE: drug-eluting embolic transarterial chemoembolization, DSM-TACE: degradable starch microsphere transarterial chemoembolization, TARE: transarterial radioembolization, SIRT: selective internal radiation therapy, Y-90: Yttrium-90, PVTT: portal vein tumor thrombosis, CP: Child–Pugh, ECOG PS: Eastern Cooperative Oncology Group Performance Status, CR: complete response, PR: partial response, OR: objective response, ORR: objective response rate, DCR: disease control rate, OS: overall survival, PFS: progression-free survival, TTP: time to progression, AEs: adverse events, TRAE: treatment-related adverse event, TRAEs: treatment-related adverse events. Note: Age reported in ‘italics’ represents median age.

**Table 3:** Summary of subgroup analysis results comparing TARE and TACE.

Subgroup		OS [HR (CI); I2]	ORR [RR (CI); I2]	PFS [HR (CI); I2]	Grade ≥ 3 AEs [RR (CI); I2]	Any AEs [RR (CI); I2]
Main analysis	-	0.99 (0.70–1.39); 88%	0.94 (0.84–1.05); 63.6%	0.54 (0.29–1.01); 88%	0.67 (0.33–1.33); 54.7%	0.91 (0.67–1.23); 68.3%
Region	Western	1.03 (0.67–1.59); 87.4%	0.96 (0.72–1.28); 76.9%	0.52 (0.23–1.21); 91.9%	0.83 (0.55–1.25); 0%	1.16 (0.85–1.59); 2%
	Non-western	0.92 (0.52–1.64); 87.2%	0.95 (0.86–1.04); 19.3%	-	0.26 (0.02–4.02); 83.3%	0.64 (0.45–0.91); 76.5%
	USA-based studies	1.01 (0.60–1.69); 60.9%	0.96 (0.69–1.34); 79.1%	-	0.87 (0.41–1.87); 0%	1.32 (0.84–2.05); 0%
Year of publication	Before 2021	0.82 (0.50–1.35); 73.4%	0.80 (0.57–1.12); 68.7%	-	0.99 (0.50–1.96); 0%	1.38 (1.03–1.86); 0%
	2021 and onwards	1.07 (0.73–1.58); 87%	1.04 (0.98–1.10); 0%	-	0.39 (0.07–2.02); 81.4%	0.66 (0.52–0.85); 31.2%
Prior treatment line	Naïve	0.84 (0.56–1.25); 47.2%	0.88 (0.69–1.14); 72.3%	-	-	-
Study design	RCT	-	-	-	0.84 (0.53–1.35); 0%	1.06 (0.65–1.74); 41.8%
	Observational	1.05 (0.74–1.51); 88.7%	0.92 (0.80–1.05); 61%	0.60 (0.28–1.26); 89.9%	0.45 (0.12–1.66); 69.1%	0.85 (0.55–1.33); 68.3%
Baseline comparability	Sufficient *	0.92 (0.67–1.25); 58.2%	0.87 (0.67–1.13); 70.4%	0.52 (0.23–1.21); 91.9%	0.55 (0.14–2.13); 75.3%	1.05 (0.69–1.58); 68%
Study quality	Low RoB	1.09 (0.74–1.60); 85.5%	0.93 (0.74–1.16); 66.7%	-	0.35 (0.07–1.77); 76.2%	1.01 (0.64–1.58); 52.3%

Abbreviations: AE: adverse event; CI: confidence interval; HR: hazard ratio; ORR: objective response rate; OS: overall survival; PFS: progression-free survival; RCT: randomized controlled trial; RoB: risk of bias; RR: risk ratio; USA: United States of America. All comparisons are TARE vs. TACE. For ORR, the comparison is TACE vs. TARE. Relative treatment effects (HR or RR) < 1 indicated that results favored TARE, whereas values >1 indicated that results favored TACE. Relative treatment effects (HR or RR) for all outcomes with 95% CIs followed by heterogeneity (I^2^) are given in the table. Empty boxes indicate that less than 3 studies were available, so meta-analysis was not conducted. For OS and PFS, HR is reported and for other outcomes RR is reported. Studies were categorized as “Western” if conducted in North America or Europe (e.g., USA, Germany, Belgium) and as “non-Western” if conducted in Asia–Pacific or Africa (e.g., China, Japan, South Korea). RoB was assessed using Cochrane Risk of Bias 2.0 tool (for RCT) and Newcastle-Ottawa scale (for observational studies). * “Sufficient control” was defined as situations where either baseline characteristics were balanced prior to adjustment techniques (no significant differences or no standardized mean differences > 10%, or where adjustment techniques (such as propensity score matching, inverse-probability of treatment weighting or multivariable linear regression) were used to mitigate imbalance successfully. Studies where baseline characteristics that may impact the decision to treat with TACE or TARE were sufficiently controlled for to mitigate confounding by indication were included for meta-analysis.

## Data Availability

The datasets produced during this study are available from the corresponding author upon request.

## Supplementary Materials

The following supporting information can be downloaded at: https://www.mdpi.com/article/10.3390/cancers18121985/s1, Figure S1: Subgroup analysis of overall survival by (A) geographic region (western and non-Western region); (B) publication era (before 2021 vs. 2021 and onwards); (C) studies with low risk of bias; (D) observational studies; (E) treatment naïve population; (F) US-based studies; (G) studies with sufficiently controlled baseline characteristics; Figure S2: Subgroup analysis of objective response rate by (A) geographic region (western and non-Western); (B) publication era (before 2021 vs. 2021 and onwards); (C) studies with low risk of bias; (D) observational studies; (E) treatment naïve population; (F) US-based studies; (G) studies with sufficient baseline control; Figure S3: Subgroup analysis of progression-free survival by (A) geographic region (western and non-Western region); (B) observational studies and (C) studies with sufficiently controlled baseline characteristics; Figure S4: Subgroup analysis of grade ≥3 adverse events by (A) geographic region (western and non-Western); (B) publication era (before 2021 vs. 2021 and onwards); (C) studies with sufficient baseline control; (D) studies with low risk of bias; (E) study design (RCT and observational); (F) US-based studies; Figure S5: Subgroup analysis of any grade adverse events by (A) geographic region (western and non-Western); (B) publication era (before 2021 vs. 2021 and onwards); (C) studies with low risk of bias; (D) study design (RCT and observational); (E) US-based studies; (F) studies with sufficient baseline control; Figure S6: Funnel plots for publication bias of (A) overall survival; (B) objective response rate; (C) progression-free survival; (D) grade 3+ AEs; (E) any AEs; Table S1: PRISMA checklist; Table S2: PubMed search strategy; Table S3: Embase search strategy; Table S4: Quality assessment of real-world evidence; Table S5: Quality assessment of RCTs.
