# Supplementary figures and images for: Transarterial Chemoembolization Versus Transarterial Radioembolization in Hepatocellular Carcinoma: A Systematic Review and Meta-Analysis of Real-World and Clinical Trial Evidence
Source: Cancers (Basel). 2026 Jun 18;18(12):1985. doi: 10.3390/cancers18121985 (PMC13297433; doi:10.3390/cancers18121985)

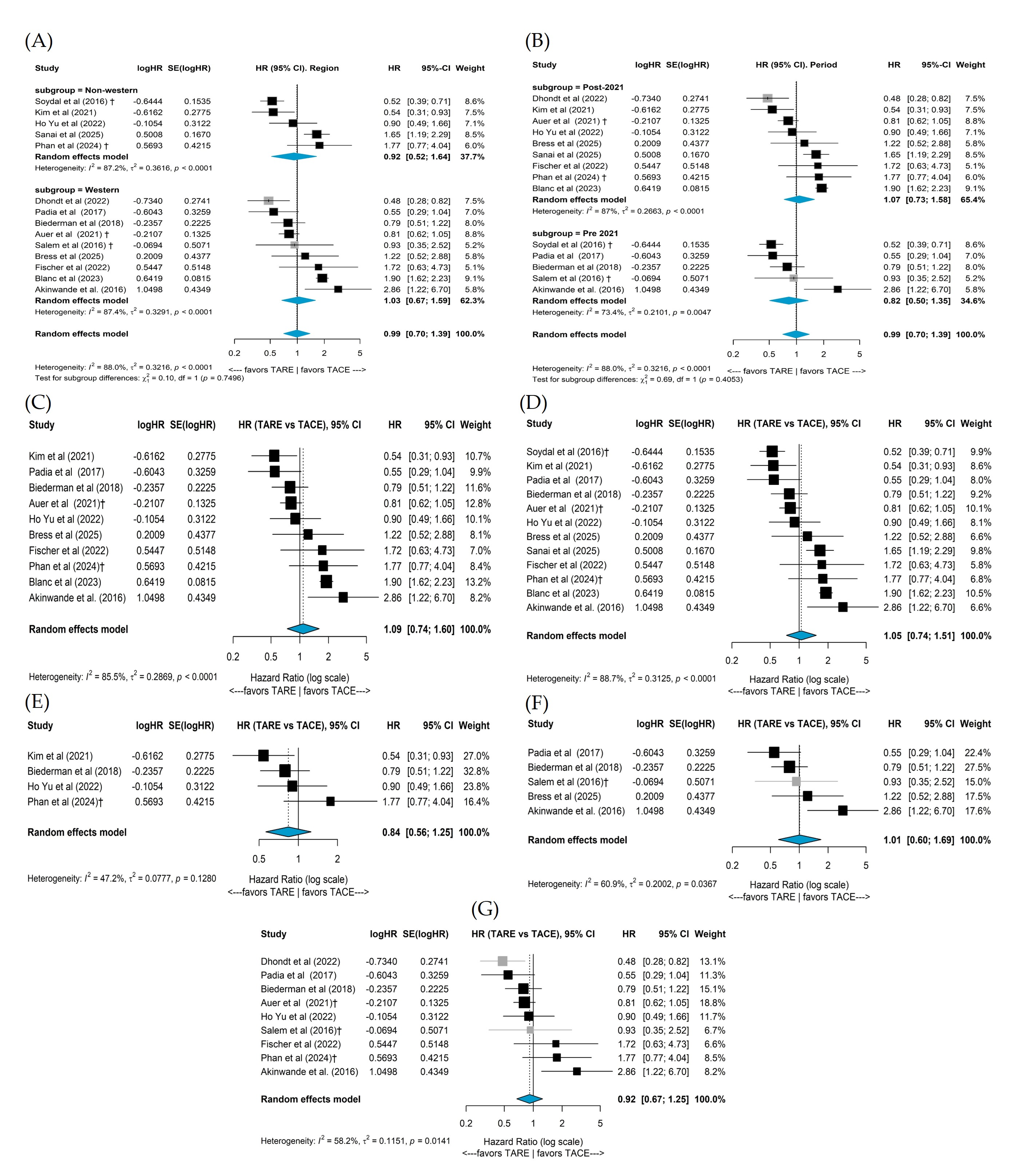

Supplement: Supplementary file 1 [file cancers-18-01985-s001.zip › Supplementary Figure S1.tiff]

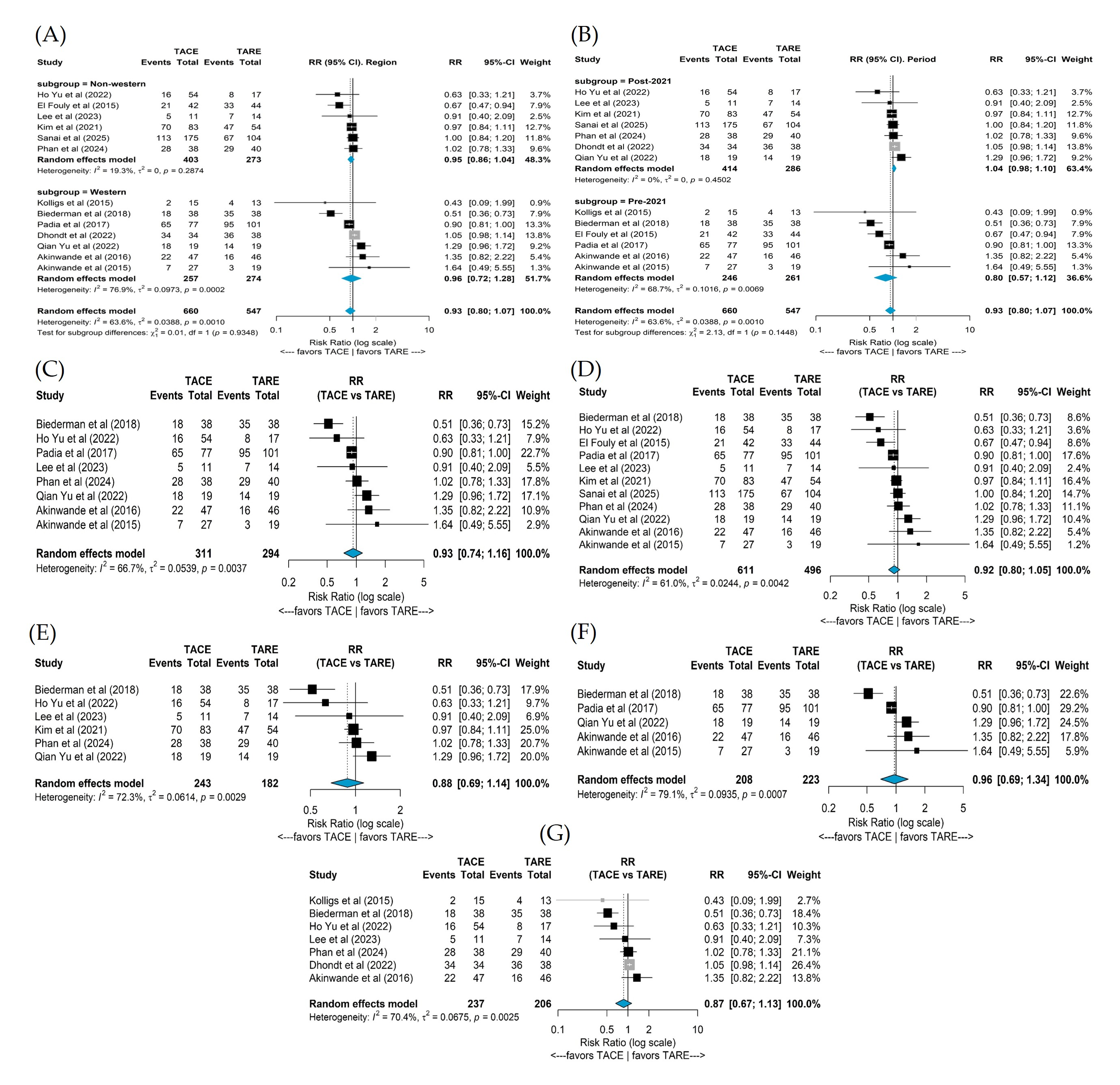

Supplement: Supplementary file 1 [file cancers-18-01985-s001.zip › Supplementary Figure S2.tiff]

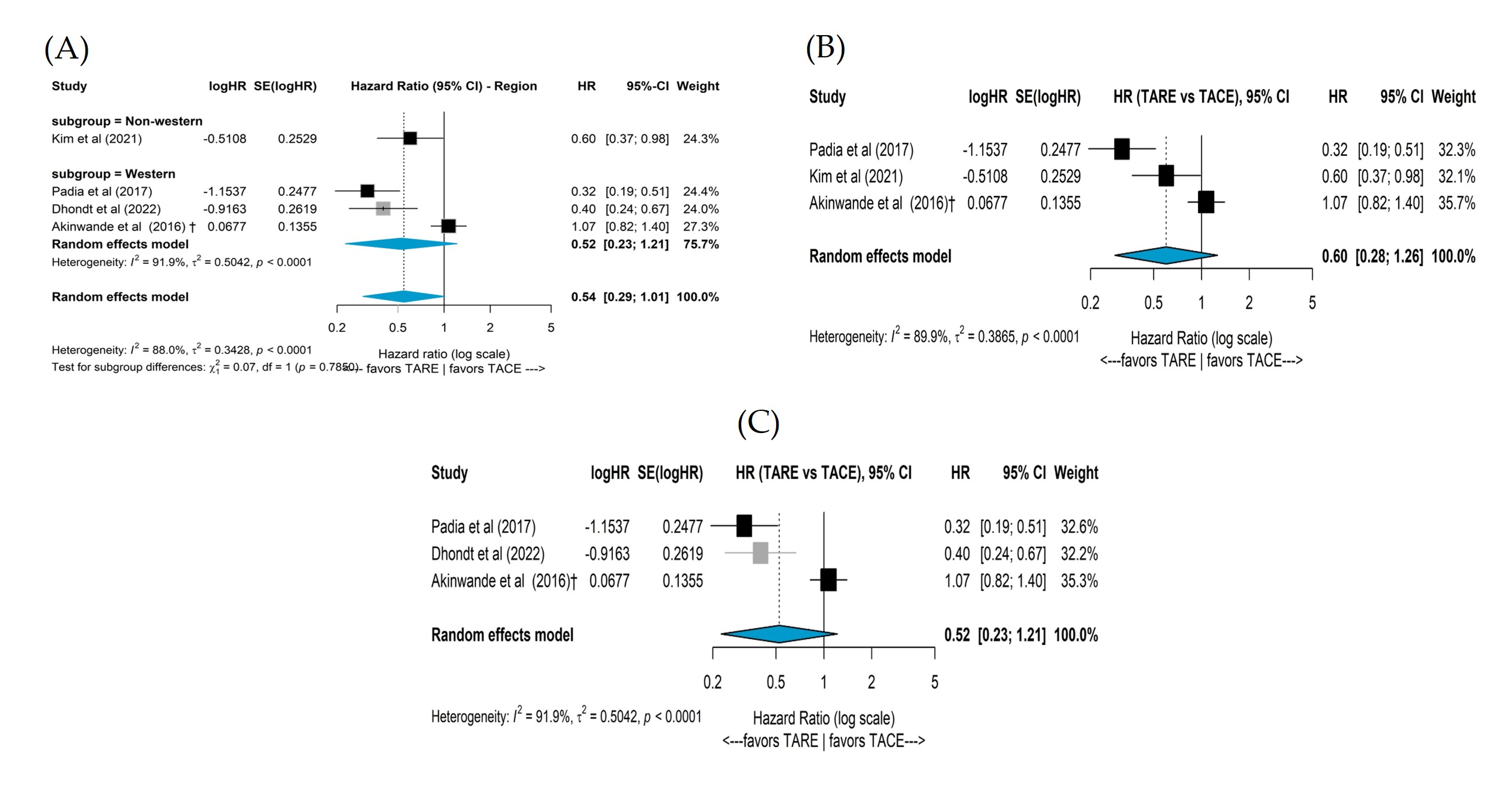

Supplement: Supplementary file 1 [file cancers-18-01985-s001.zip › Supplementary Figure S3.tiff]

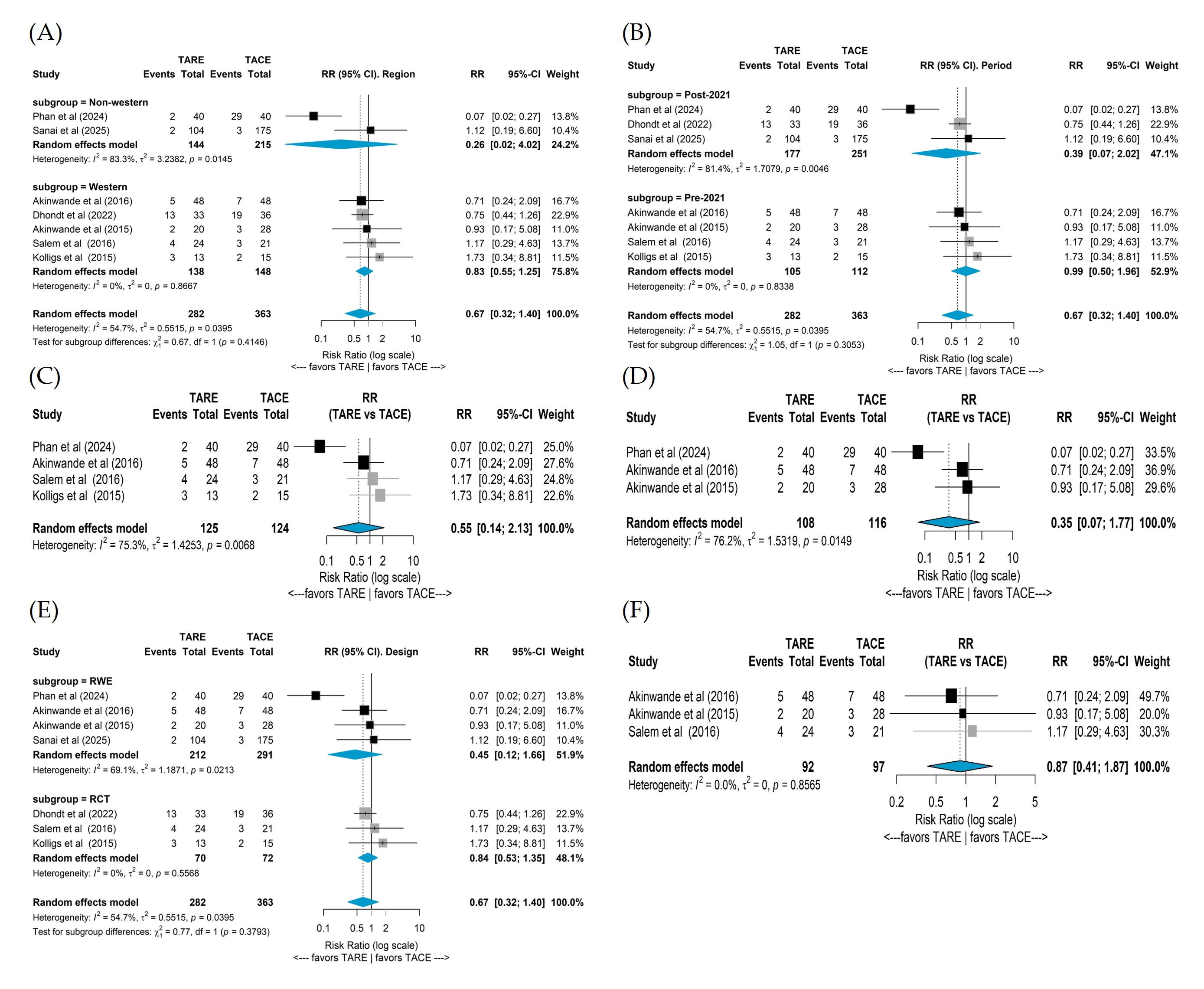

Supplement: Supplementary file 1 [file cancers-18-01985-s001.zip › Supplementary Figure S4.tiff]

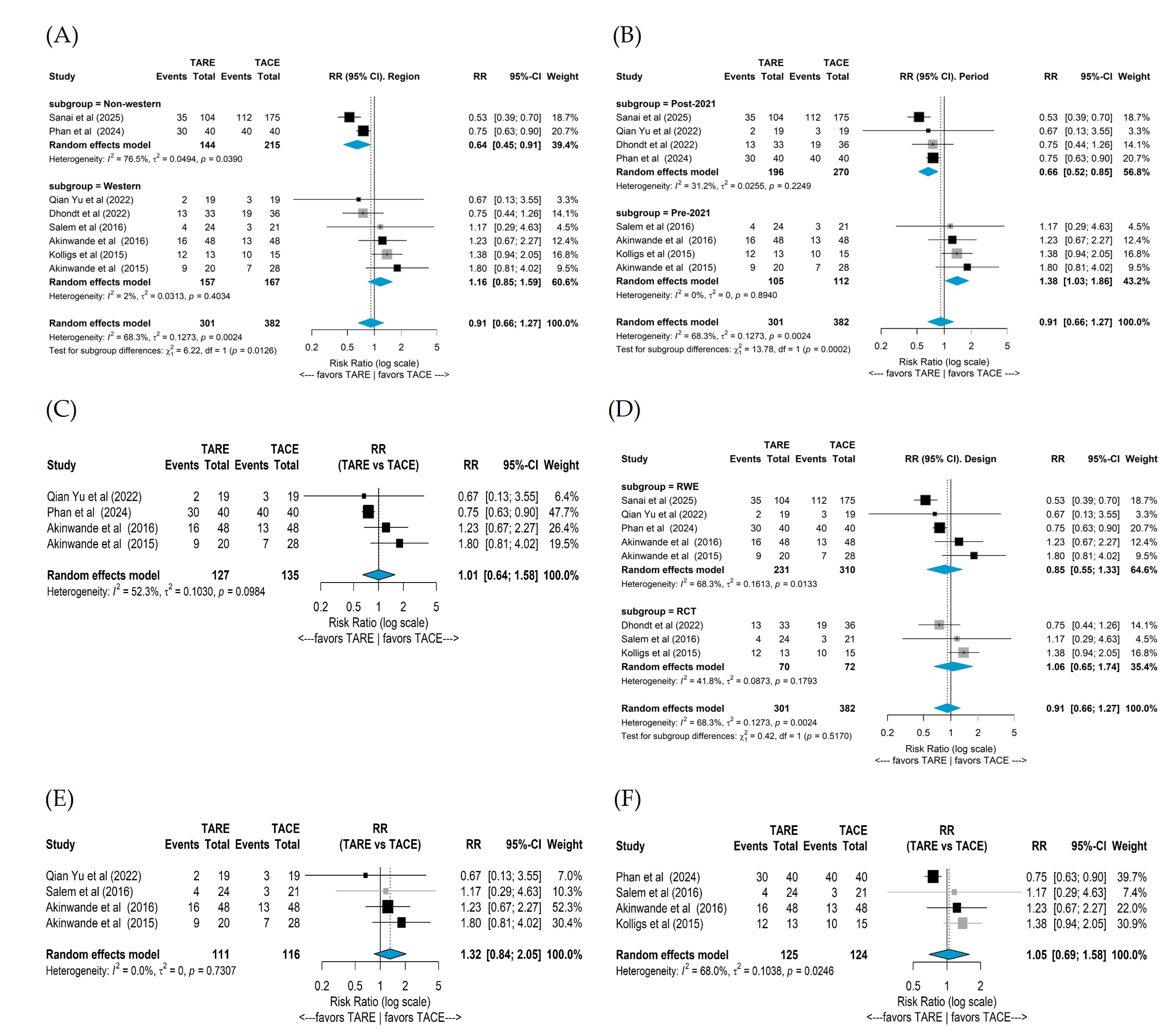

Supplement: Supplementary file 1 [file cancers-18-01985-s001.zip › Supplementary Figure S5.tiff]

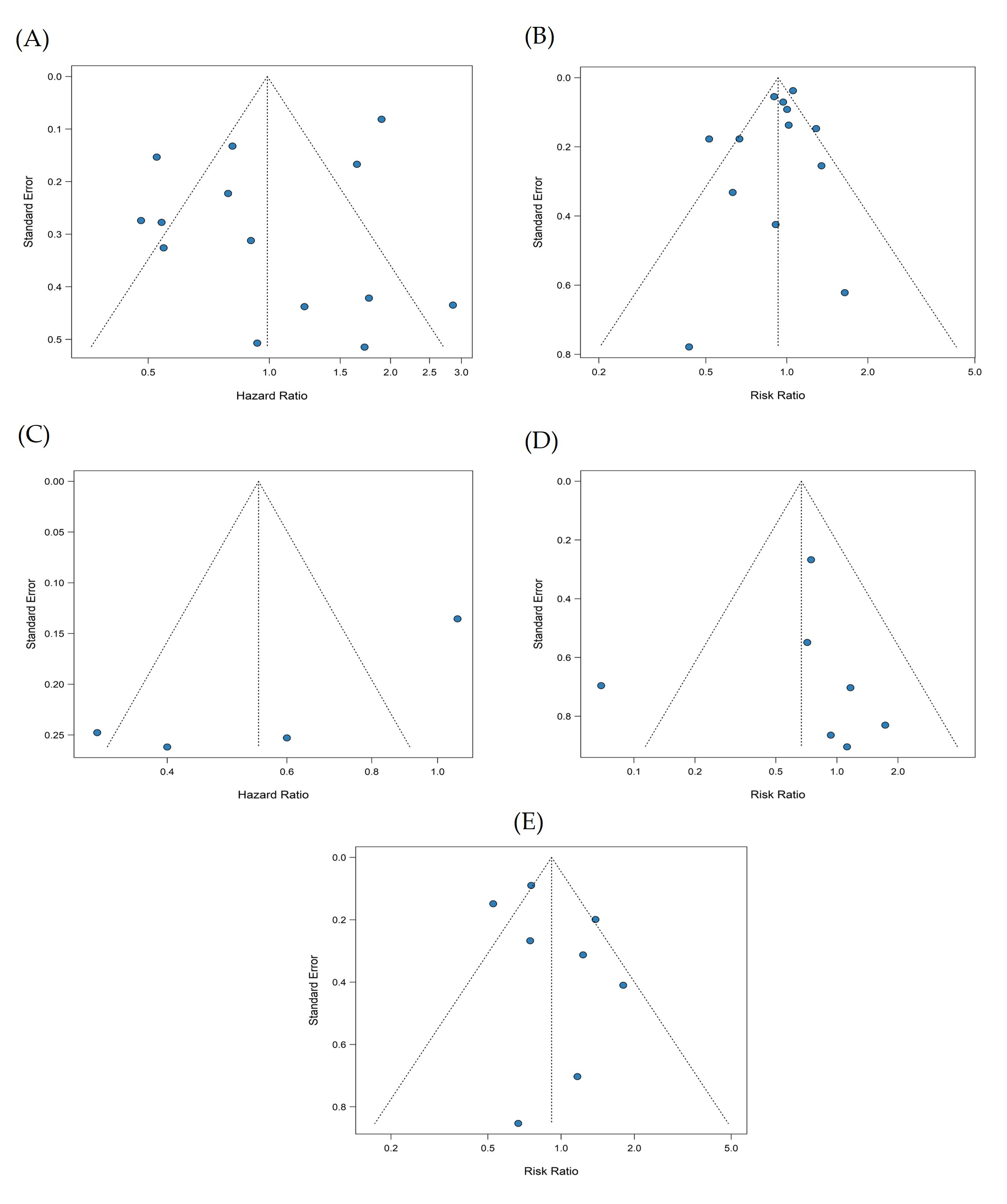

Supplement: Supplementary file 1 [file cancers-18-01985-s001.zip › Supplementary Figure S6.tiff]
